# Diffusion tensor studies and voxel-based morphometry of the temporal lobe to determine the cognitive prognosis in cases of Alzheimer’s disease and mild cognitive impairment: Do white matter changes precede gray matter changes?

**DOI:** 10.1186/s40064-016-2692-5

**Published:** 2016-07-08

**Authors:** Toshiaki Taoka, Fumihiko Yasuno, Masayuki Morikawa, Makoto Inoue, Kuniaki Kiuchi, Soichiro Kitamura, Kiwamu Matsuoka, Toshifumi Kishimoto, Kimihiko Kichikawa, Shinji Naganawa

**Affiliations:** Department of Radiology, Nagoya University Hospital, 65 Tsurumai-cho, Showa-ku, Nagoya, Aichi 466-8550 Japan; Department of Psychiatry, Nara Medical University, 840 Shijo-cho, Kashihara, Nara 634-8522 Japan; Department of Radiology, Nara Medical University, 840 Shijo-cho, Kashihara, Nara 634-8522 Japan

**Keywords:** Alzheimer’s disease, Diffusion tensor image, Voxel-based morphometry, Prognosis, Biomarker

## Abstract

**Purpose:**

The purpose of the current study was to assess the feasibility of diffusion tensor imaging (DTI) parameters for determining the prognosis of Alzheimer’s disease (AD). We also analyzed the correlation among DTI, voxel-based morphometry (VBM), and results of the mini-mental state examination (MMSE).

**Methods:**

The subjects of this prospective study were patients with AD and mild cognitive impairment. We performed annual follow-ups with DTI, VBM, and MMSE for 2 or 3 years. On DTI, the apparent diffusion coefficient (ADC) and fractional anisotropy (FA) of the uncinate fascicles were measured. VBM was performed to provide a z-score for the parahippocampal gyrus. The correlations among these factors were evaluated in the same period and the next period of the follow-up study.

**Results:**

For evaluation of the same period, both DTI parameters and z-scores showed statistically significant correlations with the MMSE score. Also for evaluation of the next period, both DTI parameters and z-scores showed statistically significant correlations with the MMSE score of the next period. We observed a statistically significant correlation between the ADC value of the uncinate fascicles and the z-score of the next period.

**Conclusions:**

Diffusion tensor parameters (ADC and FA) of the uncinate fascicles correlated well with cognitive function in the next year and seemed to be feasible for use as biomarkers for predicting the progression of AD. In addition, the white matter changes observed in the ADC seemed to precede changes in the gray matter volume of the parahippocampal gyrus that were represented by z-scores of VBM.

## Background

Pathological findings of Alzheimer’s disease (AD) include senile plaques with amyloid β deposition, neurofibrillary tangles with tau accumulation, and neuronal and glial cell apoptosis. Atrophic changes in the gray matter begin in the entorhinal cortex in the parahippocampal gyrus, followed by changes in the hippocampus, amygdala, and temporo-parieto-occipital border zone with disease progression. A large amount of research has been conducted with respect to changes in these gray matter regions (Perry et al. 2013; Yang et al. 2012). Magnetic resonance imaging (MRI) has been used to evaluate changes in the volume of the cortex, in particular, the parahippocampal gyrus including entorhinal cortex, not only in research studies but also in clinical practice (Kodama et al. 2002; Matsuda et al. 2012). On the other hand, studies on white matter changes in AD were initiated later than those of gray matter, but with the recent development of the diffusion tensor method, studies of white matter changes in AD cases with MRI are in progress (Kiuchi et al. 2009; Liu et al. 2011; Morikawa et al. 2010; Serra et al. 2012; Taoka et al. 2006, 2009; Yasmin et al. 2008; Zhang et al. 2009). Because changes in gray matter in AD as described above have been well established, changes in white matter have tended to be interpreted only as secondary changes due to the changes in gray matter (Englund 1998). Certainly, white matter fibers that contact the gray matter of the medial temporal lobe may cause Wallerian degeneration with the collapse of cells in those areas. However, whether changes in white matter are only secondary due to gray matter changes in AD remains an open question.

The purpose of the current study was to assess if changes in white matter of the limbic system predict changes in cognitive function and to evaluate the feasibility of diffusion tensor parameters as biomarkers for predicting the progression of AD. In addition, we examined the precedence between pathological changes in white matter and that in gray matter, in another word, the order of pathological changes in white matter and gray matter. We evaluated the correlation among white matter changes with the apparent diffusion coefficient (ADC) and fractional anisotropy (FA) of the uncinate fascicles, volume changes in the parahippocampal cortex with voxel-based morphometry (VBM), and results of the mini-mental state examination (MMSE) as a measure of cognitive function in cases in which follow-up MRI examination including diffusion tensor images (DTI) and volumetric images were obtained for more than 2 years.

## Methods

The subjects of this study were consecutive 35 patients with both AD and mild cognitive impairment (MCI; 13 males and 22 females; age range 58–86 years old; mean 73.2 years; median 71 years) whose last examinations were made in 2013. We performed annual follow-ups with a diffusion tensor study, morphological imaging study, and clinical study including the MMSE score for 2 or 3 years. We obtained permission from the institutional review board at our institute (http://www.naramed-u.ac.jp/university/kenkyu-sangakukan/kakushuinkaito/inorinriinkai.html). Informed consent for the imaging study was obtained from all patients or their families after the nature of the procedures had been fully explained. Clinical diagnoses of AD and MCI were based on the Diagnostic and Statistical Manual of Mental Disorders-IV criteria and examination of the cognitive status including MMSE test, clock drawing test and Alzheimer’s disease assessment scale. All patients were right-handed. The initial MMSE score of these patients was: less than 10: 2 cases, 11–20: 14 cases, 21–23: 10 cases, and more than 24: 9 cases. Of the 35 patients, 2 years of follow-up was made in 24 cases and 3 years of follow-up was made in 11 cases. Thus, the total number of studies was 81 (24 × 2 + 11 × 3). The interval between follow-ups was 11–13 months, and the imaging study and MMSE study were performed with an interval of less than 1 month.

A 1.5-T clinical MR unit (Magnetom Sonata, Siemens AG, Erlangen, Germany) was used for acquisition of DTI and T1-weighted 3D images for VBM. Diffusion-weighted images were obtained using the EPI sequence (TR = 4900 ms, TE = 85 ms, b = 1000, 6 axes encoded, averaging = 4, FOV = 230 mm, matrix = 128 × 128, number of slices = 50, slice thickness = 3 mm). Images for VBM were obtained using the T1-weighted magnetization-prepared rapid acquisition gradient echo sequence (MPRAGE: TR = 10 ms, TE = 3.5 ms, FA = 10 deg, FOV = 23, matrix = 256 × 256, 2 mm thickness).

**Fig. 1:**
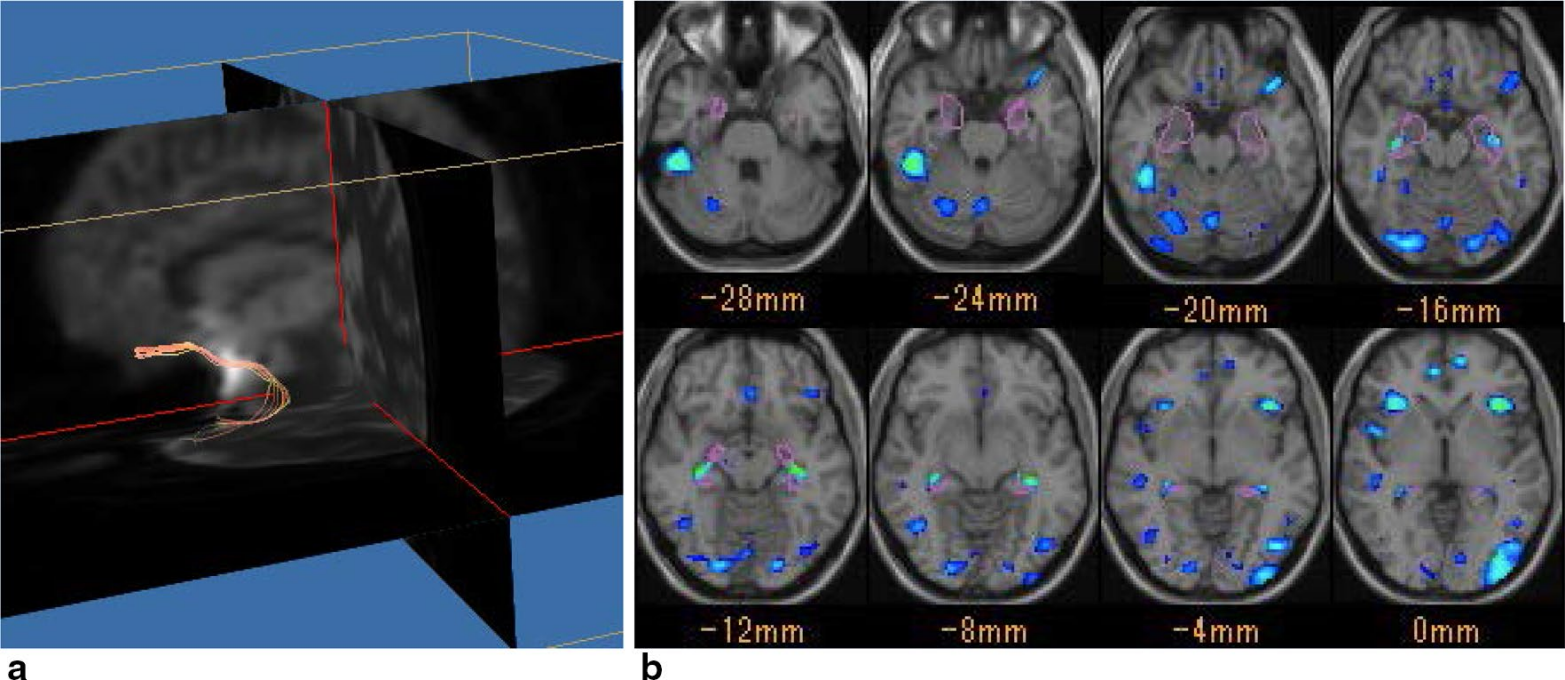
Diffusion tensor tractography of the uncinate fascicles and volume of interest of voxel-based morphometry. **a** Uncinate fascicles are visualized by using dTV II software. The FA and ADC along the tract were measured. **b** Voxel-based morphometry was performed using the Voxel-Based Specific Regional Analysis System for Alzheimer’s Disease (VSRAD). The parahippocampal gyrus is indicated as *purple lines*. The z-score of the area was calculated with this software

Diffusion tensors were computed and fiber-tract maps created on a PC workstation using the DTI software “Diffusion Tensor Visualizer ver. 2 (dTV II)” (Masutani et al. 2003). Interpolation along the z-axis was applied to obtain isotropic data (voxel size 0.89 × 0.89 × 0.89 mm). We measured mean value of FA and ADC in the whole cerebellum as global FA and global ADC. For fiber tracking, the eigenvector associated with the largest eigenvalue or the principal axis was assumed to represent the local fiber direction. The tracking algorithm moved along the principal axis. The diffusion tensor at the next location was determined from the adjacent voxels, and its principal axis was subsequently estimated. Tracking lines were traced in this way and propagated in anterograde and retrograde directions until the FA fell below an assigned threshold. The FA threshold for tracking was set to 0.18. Tractographies of the uncinate fasciculus were obtained with the seed area in the white matter of the frontal lobe on coronal planes at the tip of the frontal horn of the lateral ventricle and with a target area in the white matter on coronal planes at the tip of the inferior horn of the lateral ventricle in the ipsilateral temporal tip. A single operator drew all the tractographies in the same manner. The dTV II software has a function that calculates the mean FA and ADC (mm^2^/s) values along the tractograph. We measured the mean FA and ADC values along the bilateral uncinate fasciculus (Fig. 1a).

VBM was performed with MPRAGE images using software called “Voxel-Based Specific Regional Analysis System for Alzheimer’s Disease (VSRAD)”, which was developed by Matsuda et al. (Matsuda et al. 2012). This software uses SPM8 (Wellcome Department of Imaging Neuroscience, London, United Kingdom) to segment images into gray matter, white matter, and cerebrospinal fluid images with a unified tissue-segmentation procedure after correction for image intensity and non-uniformity. These segmented gray matter images were then spatially normalized to a customized template in the standardized anatomic space by using DARTEL (Wellcome Department of Imaging Neuroscience). Each processed gray matter image of the patients with AD and healthy controls which is provided in this software library was compared with the mean and standard deviation of gray matter images of the controls from chosen for the group comparison by using voxel-by-voxel z-score analysis with and without voxel normalization to global mean intensities (global normalization). The z-score was defined as ([control mean] − [individual value])/(control standard deviation). This program registered the target volume of interest (VOI) in the parahippocampal gyrus defined by the aforementioned group comparison, meaning that a higher z-score represents more severe atrophy in the parahippocampal gyrus (Fig. 1b) (Matsuda et al. 2012).

Thus, the datasets for each study contain FA and ADC of the uncinate fascicles on both sides, the z-score of the parahippocampal gyrus, and the MMSE score. We performed the following evaluations with these datasets.

1. Analysis of the data within the same period: We used linear regression to evaluate the correlation among six parameters (MMSE, z-score, Global FA, Global ADC, Right FA, Right ADC, Left FA, and Left ADC) of the 81 studies in the same period from 35 cases. We obtained correlation coefficients and P values for these data.
2. Analysis of the datasets compared with the next period: We also used linear regression to compare these parameters to those of the next period (MMSE, z-score, Right FA, Right ADC, Left FA, Next MMSE, Next z-score, Next Left ADC, Next right FA, Next right ADC, Next left FA, and Next left ADC) and obtained correlation coefficients and P values. Because we examined 24 cases with 2 years of follow-up and 11 cases with 3 years of follow-up, 46 datasets (24 + 11 × 2) with follow-up data for the next year were available. Specifically, correlations with the MMSE of the next period were assessed to evaluate the feasibility for predicting a decline in cognitive function by using the z-score of the parahippocampal gyrus and diffusion tensor parameters (FA, ADC) of the uncinate fascicles as biomarkers.
3. In addition, correlations between the z-score and diffusion tensor parameters of the next period, and correlations between diffusion tensor parameters and the z-score of the next period were assessed to evaluate which one is the preceding factor for the progress of the pathological changes.

## Results

**Fig. 2:**
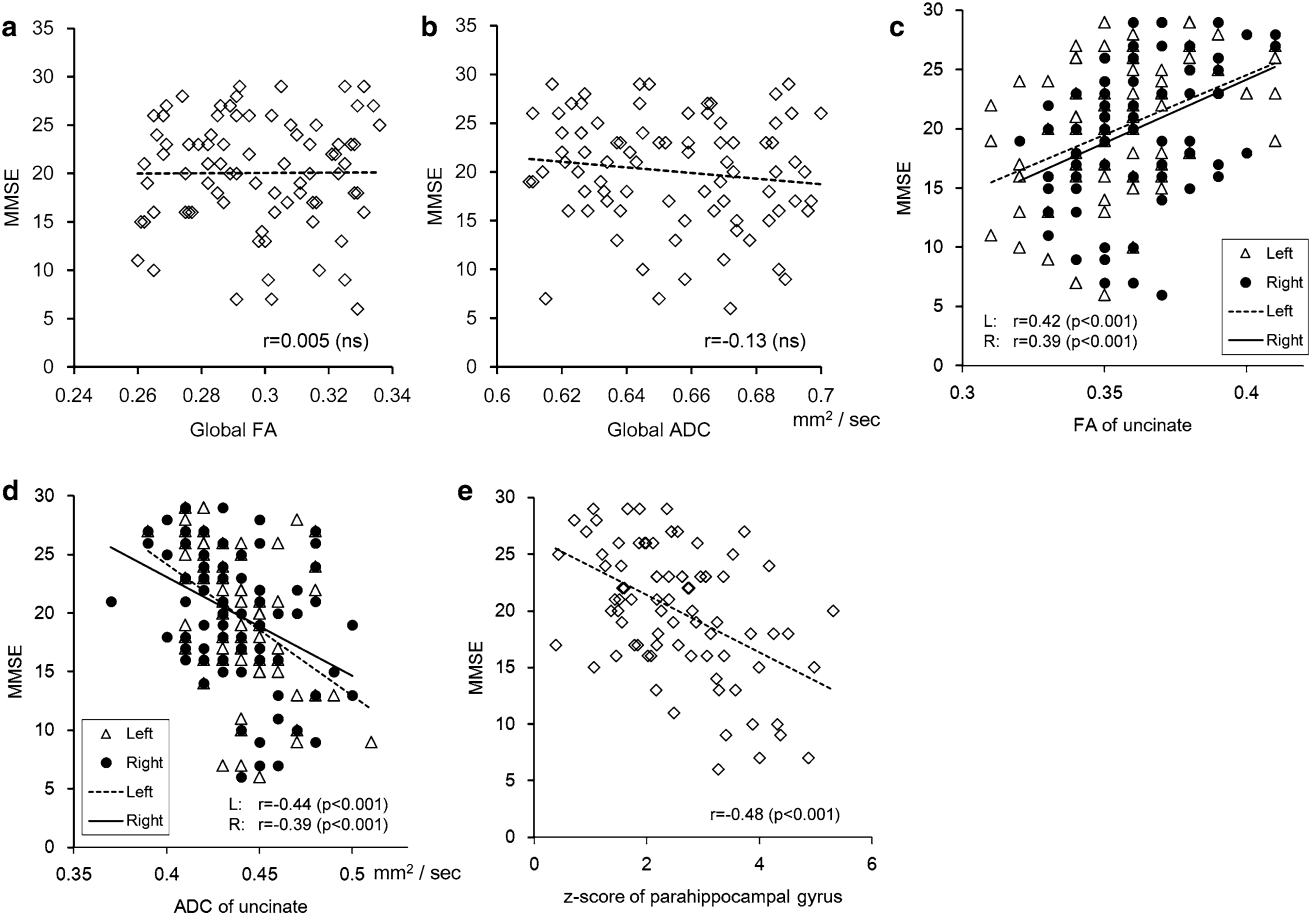
Correlations with MMSE scores in the same period. **a** Correlation between global FA and the MMSE of the same period. **b** Correlation between global ADC and the MMSE of the same period. **c** Correlation between FA of the uncinate fascicles and the MMSE of the same period. **d** Correlation between ADC of the uncinate fascicles and the MMSE of the same period. **e** Correlation between the z-score of the parahippocampal gyrus and the MMSE of the same period. Both global FA and global ADC did not shown statistically significant correlation. FA of the uncinate fascicles of *both sides* showed a positive correlation (*right* r = 0.42, *left* r = 0.39), and ADC of the uncinate fascicles of *both sides* showed a negative correlation (*right* r = −0.44, *left* r = −0.39) with the MMSE of the same period. Volume loss of the parahippocampal gyrus showed a negative correlation (r = −0.48)

The correlation coefficient and the P values among diffusion tensor parameters (FA, ADC) of the uncinate fascicles on both sides, the z-score of the parahippocampal gyrus, and the MMSE score for the same period are shown in Table 1. The correlation plots for the same period are shown in Fig. 2. Both global FA and global ADC did not shown statistically significant correlation. FA of both sides showed a statistically significant (P < 0.001) positive correlation, and ADC of both sides showed a statistically significant (P < 0.001) negative correlation with the MMSE of the same period. Volume loss in the parahippocampal gyrus showed a statistically significant (P < 0.001) negative correlation with the MMSE of the same period.

**Table 1:** Correlation among ​the MMSE score, the z-score of the parahippocampal gyrus, and the diffusion tensor parameters (FA, ADC) of the uncinate fascicles on both sides of the same periods

	MMSE	z-score	R-FA	R-ADC	L-FA	L-ADC
MMSE		−0.48	0.39	−0.39	0.42	−0.44
z-score	P < 0.001		−0.08	0.33	0.01	0.27
R-FA	P < 0.001	NS		−0.53	0.68	−0.45
R-ADC	P < 0.001	P < 0.01	P < 0.001		−0.30	0.74
L-FA	P < 0.001	NS	P < 0.001	P < 0.01		−0.41
L-ADC	P < 0.001	P < 0.05	P < 0.001	P < 0.001	P < 0.001

**Fig. 3:**
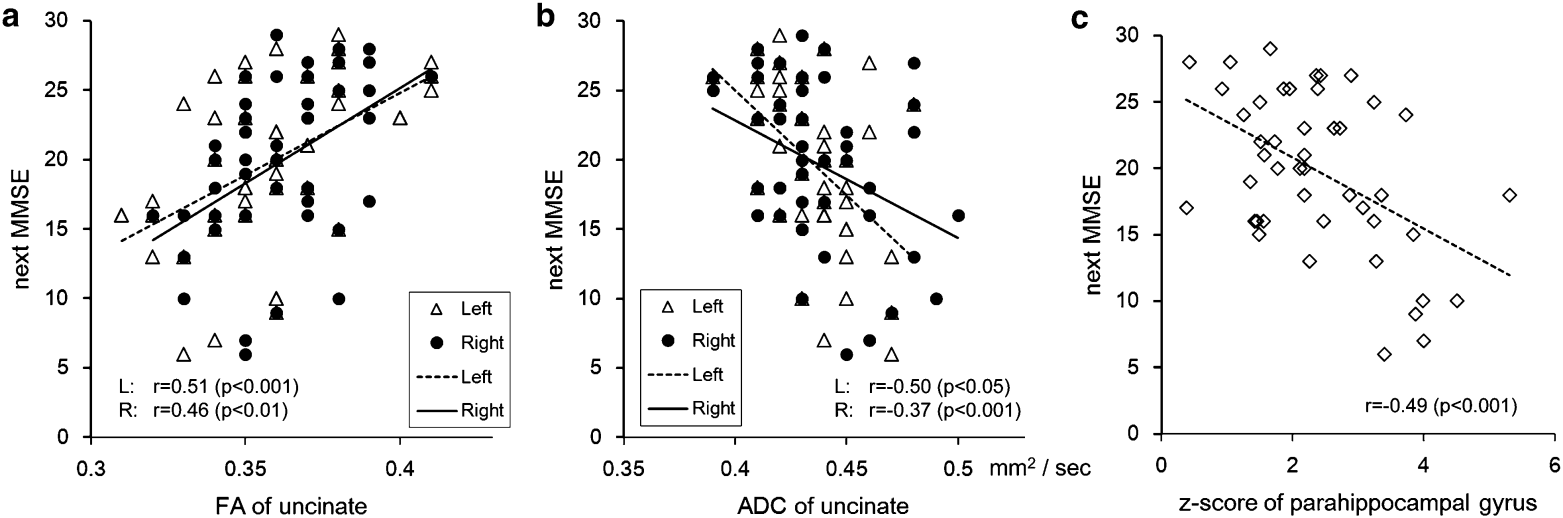
Correlations with the MMSE scores in the next period. **a** Correlation between FA of the uncinate fascicles and the MMSE of the next period. **b** Correlation between ADC of the uncinate fascicles and the MMSE of the next period. **c** Correlation between the z-score of the parahippocampal gyrus and the MMSE of the next period. FA of *both sides* showed a positive correlation (*right* r = 0.51, *left* r = 0.46), and ADC of *both sides* showed a negative correlation (*right* r = −0.50, *left* r = −0.37) with the MMSE of the next period. Volume loss of the parahippocampal gyrus showed a negative correlation (r = −0.49) with the MMSE of the next period

The correlation coefficient and the P values among diffusion tensor parameters (FA, ADC) of the uncinate fascicles on both sides, the z-score of the parahippocampal gyrus, and the MMSE score compared with the next period are shown in Table 2. The correlation plots of these parameters compared with the next period are shown in Fig. 3 (a: correlation between FA of the uncinate fascicles and MMSE of the next period, b: correlation between ADC of the uncinate fascicles and MMSE of the next period, c: correlation between the z-score of the parahippocampal gyrus and MMSE of the next period). FA on both sides showed a statistically significant (P < 0.01) positive correlation, and ADC on both sides showed a statistically significant (right: P < 0.01, left: P < 0.001) negative correlation with MMSE of the next period. Volume loss in the parahippocampal gyrus showed a statistically significant (P < 0.01) negative correlation with the MMSE of the next period.

**Table 2:** Correlation among the MMSE score, the z-score of the parahippocampal gyrus, diffusion tensor parameters (FA, ADC) of the uncinate fascicles on both sides compared with those of the next periods

	MMSE	z-score	R-FA	R-ADC	L-FA	L-ADC	Next MMSE	Next z-score	Next R-FA	Next R-ADC	Next L-FA	Next L-ADC
MMSE		−0.42	0.40	−0.38	0.49	−0.45	0.87	−0.47	0.29	−0.40	0.28	−0.39
z-score	P < 0.01		−0.04	0.35	0.09	0.34	−0.49	0.88	−0.08	0.27	−0.03	0.16
R-FA	P < 0.01	NS		−0.49	0.67	−0.36	0.46	−0.05	0.56	−0.35	0.48	−0.33
R-ADC	P < 0.01	P < 0.05	P < 0.001		−0.25	0.74	−0.37	0.46	−0.33	0.76	−0.31	0.70
L-FA	P < 0.001	NS	P < 0.001	NS		−0.35	0.51	0.07	0.52	−0.28	0.55	−0.25
L-ADC	P < 0.01	P < 0.05	P < 0.05	P < 0.001	P < 0.05		−0.50	0.39	−0.47	0.78	−0.31	0.81
Next MMSE	P < 0.001	P < 0.001	P < 0.01	P < 0.05	P < 0.001	P < 0.001		−0.52	0.32	−0.38	0.32	−0.42
Next z-score	P < 0.001	P < 0.001	NS	P < 0.01	NS	P < 0.01	P < 0.001		−0.05	0.37	−0.02	0.27
Next R-FA	NS	NS	P < 0.001	P < 0.05	P < 0.001	P < 0.01	P < 0.05	NS		−0.51	0.67	−0.46
Next R-ADC	P < 0.01	NS	P < 0.05	P < 0.001	NS	P < 0.001	P < 0.01	P < 0.05	P < 0.001		−0.31	0.78
Next L-FA	NS	NS	P < 0.001	P < 0.05	P < 0.001	P < 0.05	P < 0.05	NS	P < 0.001	P < 0.05		−0.39
Next L-ADC	P < 0.01	NS	P < 0.05	P < 0.001	NS	P < 0.001	P < 0.01	NS	P < 0.01	P < 0.001	P < 0.01

**Fig. 4:**
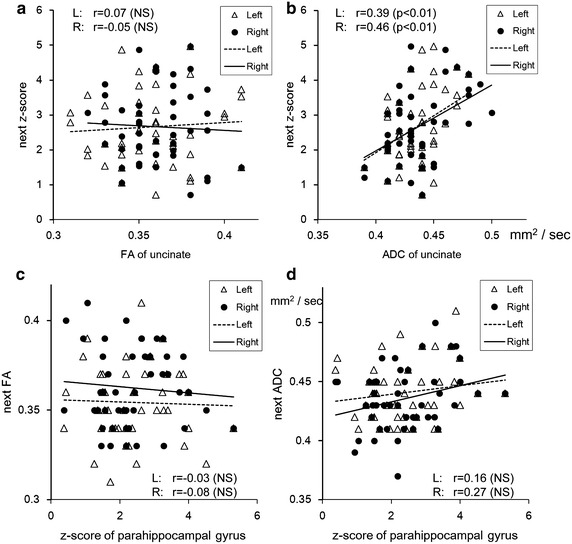
Correlation between DTI parameters and VBM scores: comparison with the next period. **a**, **b** Correlation between diffusion tensor parameters (**a** FA, **b** ADC) of the uncinate fascicles and the z-score of the parahippocampal gyrus of the next period. **c**, **d** Correlation between the z-score of the parahippocampal gyrus and diffusion tensor parameters (**c** FA, **d** ADC) of the uncinate fascicles of the next period. A significant correlation was found only in the comparison of ADC of the uncinate fascicles and the z-score of the parahippocampal gyrus of the next period (**b**) (*right* r = 0.39, *left* r = 0.46)

Figure 4a, b shows the correlation between diffusion tensor parameters of the uncinate fascicles and the z-score of the parahippocampal gyrus of the next period, and Fig. 4c, d shows the correlation between the z-score of the parahippocampal gyrus and diffusion tensor parameters of the uncinate fascicles of the next period. These results also included in Table 2a, b. We found a statistically significant (P < 0.01) correlation only in the comparison of ADC of the uncinate fascicles and the z-score of the parahippocampal gyrus of the next year.

## Discussion

AD results in progressive degeneration of the gray and white matter. The gray matter of the parahippocampal gyrus is impaired in the early stage of AD, and as the disease progresses, other regions of cerebral gray matter become impaired. The pathological changes in white matter reported in AD include the breakdown of axons or myelin sheaths and gliosis (Warren et al. 2005; Terada et al. 2004). Progression of these microstructural changes leads to a decrease in the white matter volume or changes in the water content of the white matter, and these changes lead to detectable signal changes on conventional MRI including T1- or T2-weighted images. In a study with 3D-T1-weighted imaging, a decrease in the white matter volume was reported in the lower parietal lobe and rostral middle frontal gyrus as well as the parahippocampal gyrus and entorhinal cortex (Salat et al. 2009). Results of the United States-Alzheimer’s Disease Neuroimaging Initiative study showed that the volume of the area with a high signal in the white matter on T2-weighted images can predict transition from normal to MCI (Carmichael et al. 2010). Several studies have indicated that abnormal white matter signals on images can be used to determine the prognosis of dementia (Smith et al. 2008; Guzman et al. 2013). In contrast to analysis with the above-mentioned conventional MRI, which reflects rather late, macroscopic changes in the white matter, the diffusion tensor method may reflect early pathological changes in the tissue microstructure such as alterations in myelin sheaths and axons (Huang et al. 2007). A report by Rose et al. (2000) appears to be the first to apply the diffusion tensor method in AD. The report showed that diffusion anisotropy decreases in regions of interest in the splenium of the corpus callosum, cingulum, and superior longitudinal fasciculus in cases with AD. Another study applied tract-based analysis of diffusion tensor parameters in the uncinate fasciculus, which is a limbic system circuit (Fig. 1) (Taoka et al. 2006). In this report, white matter tracts passing through the temporal stem were identified with diffusion tensor tractography to measure anisotropy and diffusivity. In the AD cases, decreased FA and increased diffusivity were observed in the limbic system tract including the uncinate fascicles.

In the current study, we selected uncinate fascicles for evaluation of white matter changes with diffusion tensor studies. The uncinate fasciculus is a part of the Yakovlev circuit, which connects the orbital gyrus in the frontal lobe and the anterior part of the temporal lobe. The Yakovlev circuit is a part of the limbic system and is thought to be related to emotion or motivation. The uncinate fasciculus is considered to play a role in cognitive and memory function. In addition, diffusion tensor tractography of the uncinate fascicles is easy to perform, and measurements are stable. Thus, several studies have assessed these fascicles and have shown correlations with disease severity of AD (Kiuchi et al. 2009; Serra et al. 2012; Taoka et al. 2006; Yasmin et al. 2008; Taoka et al. 2009). In the results of current study, evaluation of the correlation in the same period indicated that both DTI parameters (FA, ADC) of uncinate fascicles and z-scores had statistically significant correlations with the MMSE score while both global FA and global ADC did not shown statistically significant correlation. These results agree with existing studies in which both gray matter and white matter changes can be observed and are correlated with clinical symptoms including results of the MMSE (Yang et al. 2012; Carmichael et al. 2010; Baxter et al. 2006). Analyzing DTI parameters on the uncinate fascicles as well as VBM of the parahippocampal gyrus may be feasible as a biomarker, because the current study analyzed follow-up examinations performed in the next period, and both DTI parameters and z-scores showed statistically significant correlations with the MMSE score.

Several studies have shown that the white matter changes in AD are independent of cortical changes. In a study on regional patterns of white matter tissue changes in cases with AD using the diffusion tensor method, alterations in diffusion properties were found in several regions in AD including the parahippocampal white matter, which has direct and secondary connections to the medial temporal lobe (Stebbins and Murphy 2009). Diffusion tensor changes measured in the parahippocampal white matter are independent of gray matter degeneration when measuring hippocampal volume, demonstrating distinct zones of alterations that may stem from differences in the underlying pathology and potential myelin-specific pathology in the parahippocampal white matter (Salat et al. 2010). A pathological study showed the disappearance of myelin sheaths, axons, and oligodendrocytes as well as reactive gliosis in the white matter of AD. In addition, hyaline membrane degeneration of arterioles was seen. The distribution of these pathological changes is independent of the distribution of gray matter lesions (Brun and Englund 1986).

The current study also provided some information about the order of pathological changes in white matter and gray matter. The reports above indicated that the white matter changes are not sequential after gray matter changes in AD. Thus, whether the cortex or white matter degenerates first remains an unanswered question. In cases of MCI, even at a stage in which gray matter atrophy is limited to the medial part of the temporal lobe, white matter changes observed with DTI are not limited to the medial temporal lobe but are observed throughout a wide range in the brain and are independent of cortical atrophy (Agosta et al. 2011). A pathological study compared advanced cases of AD and cases before the onset of symptoms and showed remarkable atrophy in the white matter even though cortical lesions were not yet visible. The report stated that changes in the white matter are peculiar to AD and include axonal disappearance before cortical changes. Thus, white matter changes may cause cortical changes (de la Monte 1989). The current study showed that white matter changes in the uncinate fascicles represented by DTI parameters were correlated with gray matter changes in the next period as observed with the z-score of the parahippocampal gyrus. We also evaluated if gray matter changes as observed with the z-score were correlated with white matter changes represented by DTI in the next period. We found that only the ADC value of uncinate fascicles was correlated with the z-score in the next year, and other factors including the FA value or z-score value were not significantly correlated with the z-score or DTI parameters in the next year. Of course, this finding does not demonstrate a causal relationship, but only a correlation. However, these results support the idea that white matter changes precede gray matter changes in the pathological progress of AD.

There are several limitations in the current study. First, the small sample size is rather small. In addition, we applied a 6-axis diffusion encoding gradient, which is a small number for diffusion encoding. Because the AD subjects in this study had problems in their cognitive functions, we needed to set shorter imaging time by using smaller number of diffusion encoding. Another limitation is that our analysis was limited to uncinate, a part of cingulate. There are other tracts which reported show pathological changes including cingulum or inferior occipito-frontal fascicles. Since we are using tract based analysis, comparison of multiple tract may lead to confusion, so we selected only uncinate for evaluation. We did not applied multiple comparison correction technique so the comparison might be suffered from possibility of look-elsewhere error. While the data was acquired non-isoptropically which is not ideal for DTI and tractography analysis, we did not validate our findings in a separate data.

In conclusion, diffusion tensor parameters (FA, ADC) for the uncinate fascicles showed statistically significant correlations with the MMSE at the time of imaging and with the MMSE in the next year. These parameters of the uncinate fascicles correlated well with the cognitive status in the next year, and thus can be used as biomarkers to predict AD progression and volumetric parameters of the parahippocampal gyrus. In addition, the white matter changes shown with ADC of DTI seemed to precede the changes in the gray matter volume as represented by the z-score of VBM in the parahippocampal gyrus, since there was a statistically significant correlation in the comparison of ADC of the uncinate fascicles and the z-score in the parahippocampal gyrus in the next year.
